# Riding the Adolescence: Personality Subtypes in Young Moped Riders and Their Association With Risky Driving Attitudes and Behaviors

**DOI:** 10.3389/fpsyg.2019.00300

**Published:** 2019-02-18

**Authors:** Fabio Lucidi, Luca Mallia, Anna Maria Giannini, Roberto Sgalla, Lambros Lazuras, Andrea Chirico, Fabio Alivernini, Laura Girelli, Cristiano Violani

**Affiliations:** ^1^Department of Social and Developmental Psychology, Sapienza University of Rome, Rome, Italy; ^2^Department of Movement, Human and Health Sciences, University of Rome “Foro Italico”, Rome, Italy; ^3^Department of Psychology, Sapienza University of Rome, Rome, Italy; ^4^Department of Public Security, Ministry of Interior, Rome, Italy; ^5^Department of Psychology, Sociology and Politics, Sheffield Hallam University, Sheffield, United Kingdom; ^6^National Institute for the Evaluation of the Education System, Rome, Italy; ^7^Department of Human, Philosophical, and Educational Sciences, University of Salerno, Fisciano, Italy

**Keywords:** moped riders, personality, attitudes toward safety, risky driving behavior, cluster analysis

## Abstract

The aim of the present study was to identify sub-types of moped riders based on a cluster analysis of specific personality characteristics (i.e., driving anger, anxiety, angry hostility, excitement-seeking, altruism, normlessness, and driving locus of control) within a large sample of Italian adolescents. The study had also the aim to compare the emerged sub-types of moped riders on measures of attitudes toward safe driving, risky driving behaviors (e.g., rule’s violations and speeding, not using helmet, drinking and driving, etc.), and self-reported tickets and accident involvement. One thousand two hundred seventy-three Italian high school students aged from 13 to 19 years (mean_age_ = 15.43, *SD* = 0.98) with a valid driving license for moped participated to the study. Results revealed three sub-types of moped riders (namely risky, worried and careful moped riders), which differ significantly for risky driving behaviors, attitudes toward traffic safety, risk perception, and self-reported accident involvement. Importantly, the results of the present study showed that the personality and behavioral characteristics of the three sub-groups of moped riders substantially resembled those identified by previous studies with vehicle drivers of different ages; thus, empirically supporting the notion that certain combinations of personality characteristics are associated with risk driving tendencies and behaviors in both young moped riders and car drivers. Safe driving interventions can tackle risky driving beliefs and behavioral tendencies in young moped riders and car drivers by tailoring their messages according to the personality sub-types of the target groups.

## Introduction

According to the World Health Organization, about 1.25 million people die in traffic crashes every year, and road traffic injuries represent the main cause of death among young adults between 15 and 19 years old (World Health Organization [WHO], 2018). The use of mopeds (50 cc and restricted top speed) or motorcycles, has increased over the last 15 years, especially among younger people and adolescents living in dense population areas and especially in Southern European countries, such as Italy and Greece (Theofilatos and Yannis, 2015). In Italy, for example, the 57.1% of the young people aged 15–24 drives habitually a moped, with about 6,5 million of moped circulating across the country (Censis, 2003). Despite providing an economic means of transportation, the use of mopeds and motorcycles accounts for a substantial proportion of road fatalities, and moped users have increased frequency and severity of traffic crashes (Vlahogianni et al., 2012). In particular, as of 2015, moped riders and motorcyclists represented 9% of all road fatalities in the EU and nearly a quarter (i.e., 23%) of world’s fatal traffic injuries (World Health Organization [WHO], 2015). In Italy, moped drivers and motorcyclists represent about the 3% and the 20%, respectively, of the overall victims due to road accidents (ISTAT, 2018). Blackman and Haworth (2013) further argued that mopeds and motorcycles riders have 20–40 times higher risk for road fatalities as compared to car occupants. Accordingly, Brandau et al. (2011) stated that adolescent moped riders are at higher risk for traffic road injuries, and that the percentage of 15-year-old moped riders injured in traffic crashes in Austria increased from 6 to 32% between 2000 and 2008. The Decade of Action for Road Safety (DARS) 2011–2020 represents an international initiative led by the United Nations aimed to improve road safety and to reduce by 50% the number of deaths attributed to traffic injuries and crashes, especially among groups at higher risk for road traffic fatalities, such as young people. One of the key action areas of the global plan to achieve the DARS 2011–2020 goals concerns road users’ behavior (UN Road Safety Collaboration, 2011). This indicates that a better understanding of the behavioral risk factors for traffic crashes can help in further promoting road safety, particularly in the most vulnerable groups of road users, such as young moped riders.

The extant research on the behavioral and psychological risk factors for traffic crashes among young car drivers has highlighted the role of personality traits using both bivariate analysis and more sophisticated data analytic approaches, such as structural equation modeling. In particular, in the “personality-attitudes-behavior” model, Ulleberg and Rundmo (2003) hypothesized that some general personality traits of drivers such as anxiety, excitement-seeking, hostility, altruism, and normlessness are relevant for driving behavior and they could also influence risk driving trends both directly and indirectly through their effects on attitudes toward traffic safety. This model considers the personality as a distal and stable predictor of behavior, as compared to more immediate and malleable antecedents of behavioral intention and the beginning of an action such as attitudes. Attitudes, in turn, are considerated to mediate the personality-behavior relationship (Fishbein and Cappella, 2006). In their study on young Norwegian drivers, Ulleberg and Rundmo (2003) showed that most of the personality traits included in their model (i.e., anxiety, hostility, normlessness, excitement-seeking and aggression) were indirectly associated with risky driving through their effects on attitudes toward driving safety, while altruism was directly associated with risky driving. More specifically, the results of this study showed that normlessness, excitement-seeking and lack of emotional regulation – expressed through aggression – negatively affected attitudes toward safety, so these traits indirectly increased risky driving. Conversely, anxiety positively influenced attitudes and indirectly decreased the frequency of risky driving. Finally, altruism seemed to affect negatively and directly risky driving. Overall, these empirical evidences frame a clear pattern of relationships linking young drivers’ specific personality traits with their attitudes and risky driving behaviors. These patterns suggest that for young drivers having higher levels of normlessness, excitement seeking and low emotional stability and regulation (i.e., high levels of aggression) may represent a risk factor since it seems to increment risky driving behaviors, inhibiting pro-safety attitudes. On the other hand, having higher levels of anxiety and altruism, may be considered a protective factor, as it seems to decrease risky driving behaviors, enhancing pro-safety attitudes.

Interestingly, a related but different line of research has focused on clustering different personality traits that increase the likelihood for risky driving and crash risk among young car drivers (e.g., Donovan et al., 1988; Deery and Fildes, 1999; Ulleberg, 2001; Lucidi et al., 2010). This line of research adopted a clustering approach whereby young car drivers are classified as high or low risk for crashes based on patterns of personality traits and individual differences. In an early study, Ulleberg (2001) used a cluster analysis of personality traits and found that six sub-types of risky car drivers emerged. Of them, two high-risk groups were identified, with the first group including drivers with higher scores in sensation seeking, irresponsibility, and driving-related aggression, and low rates of altruism and anxiety; the second group included drivers with high levels of sensation-seeking, driving-related aggression, anxiety and driving anger. Two low risk groups also emerged. The first one included drivers with higher levels of anxiety and altruism, and lower scores in sensation-seeking, driving anger, and normlessness, and the second low risk group included drivers with low levels of sensation-seeking, anxiety, aggression, and driving anger; thus, representing an emotionally well-adjusted group. In a subsequent study among Italian young novice car drivers, Lucidi et al. (2010) performed a cluster analysis of personality traits and identified three distinct groups: risky, worried, and careful drivers. Risky drivers had higher scores in normlessness, excitement-seeking, driving anger, and external locus of control (i.e., attributing traffic crashes to external factors, such as bad luck), and lower scores in altruism and anxiety. Risky drivers had also more negative attitudes toward safe driving, had engaged in more risky driving, were more likely to be involved in crashes, and perceived themselves as less susceptible to traffic crashes as compared to drivers in the other groups. On the other hand, worried drivers had higher scores in anxiety, angry hostility, external locus of control and driving anger and lower scores in excitement-seeking, normlessness and altruism – thus, although this group may follow traffic rules and abstain from risky driving behavior, they seem to pay less attention to others, to be more emotionally unstable, and more likely attribute crashes to external factors. In addition, worried drivers displayed more positive attitudes to safe driving than risky drivers but as many lapses as them. Finally, careful drivers displayed lower scores in normlessness, driving anger, anxiety, angry hostility, and excitement-seeking, and higher scores in altruism. Those drivers also displayed higher internal locus of control in driving; indicating their beliefs that to a greater extent, traffic crashes ascribed to drivers’ behavior rather than to external causes. Careful drivers had also more positive attitudes toward safe driving, as compared to risky drivers; additionally they were less likely to be involved in a crash, and displayed less risky driving patterns, such as violations, errors, and lapses compared to risky and worried drivers.

In addition, other studies have shown that the association between personality, traffic safety attitudes, and risky driving can also be observed among young moped and motorcycle riders (Chen, 2009; Gianfranchi et al., 2017). Brandau et al. (2011), for instance, applied the cluster analysis approach to identify personality sub-types among young Austrian moped riders, aged between 14 and 17 years, and four distinct groups (Types) emerged. The first group (Type 1) had high levels of neuroticism, and low scores in extraversion and openness to experiences, Type 2 moped riders had high scores in risk taking and extraversion. Type 3 moped riders had low levels in several personality traits and psychological characteristics, including novelty seeking, risk-taking, reward dependence, inattention and impulsivity, and high rates in conscientiousness, agreeableness, and openness. Finally, the moped riders in Type 4 had higher scores in novelty seeking, risk-taking, reward dependence, inattention and impulsivity, and low rates in conscientiousness, agreeableness and openness. Brandau et al. (2011) also found that Type 3 moped riders had significantly less traffic injuries as compared to other Types, and that Type 4 moped riders had the highest rate of severe injuries. Marengo et al. (2012) assessed moped driver sub-types based on other personality measures in a sample of Italian adolescents aged 14–15 years, and they identified three clusters. Cluster A included mostly female adolescents with higher scores in anxiety, external locus of control and lower levels of sensation-seeking and altruism. Cluster B consisted of adolescents with high scores in impulsivity and sensation-seeking, and lower scores in altruism and anxiety; this group also displayed the greatest crash involvement in a riding simulator and displayed higher crash risk in relevant self-reported measures. Finally, adolescents in Cluster C displayed higher scores in altruism and internal locus of control, and were considered as the group with the least risk for traffic crashes/injuries. The study by Marengo et al. (2012) provided useful findings and also included a moped riding simulator to predict real-life crash risk. However, more than half (54%) of the adolescents in the study had no prior moped riding experience and this limited the external validity of the study.

### The Present Study

The present study is in line with the remit of the United Nations Global Plan for the DARS 2011–2020 and responds to the need to better understand road users’ behavior, especially among adolescent moped riders who represent a high-risk group for road traffic injuries and fatalities (Vlahogianni et al., 2012). Following from previous research on adolescent moped riders in Austria (Brandau et al., 2011) and Italy (Marengo et al., 2012) the present study set out to assess, in a large and representative sample of Italian adolescent moped riders, sub-types based on personality traits, and compare the different sub-types on measures of attitudes toward safe driving, and risky driving behaviors. Our study advances previous research on moped riders personality and risky driving in the following respects. Firstly, a large and representative sample of adolescents with a valid moped-riding license was used. Secondly, a wider range of measures of personality and risky driving-related outcomes (e.g., attitudes toward safe driving, risky driving behaviors) was included. Using an extensive set of measures of personality, safe driving attitudes and risky driving enabled previous research (i.e., Lucidi et al., 2010) to make indirect comparisons of risky driving psychological characteristics and behaviors among users of different types of vehicles, and allowed us to assess if previous findings could be usefully applied in adolescent moped riders. Based on the previous literature (i.e., Lucidi et al., 2010; Marengo et al., 2012) we expect three different personality subtypes of young moped drivers from the present study results, characterized also by both their attitudes toward safety and their risky driving behaviors. In particular, we expect a first cluster of moped riders, characterized mainly by high levels of excitement-seeking and normlessness, high levels of emotional instability (i.e., high driving anger and anger hostility), along with low levels of anxiety, altruism and driving internality. According to the “personality-attitudes-behavior” model introduced by Ulleberg and Rundmo (2003) we expect that these riders would be characterized as an high-risk group, showing low levels of attitudes toward safety and accidents risk perception, frequent self-reported risky driving behaviors as well as an high involvement in car accidents. Furthermore, we expect a second group of moped riders characterized by an opposite personality profile, showing low levels of excitement-seeking and normlessness, low levels of emotional instability (i.e., low anger and angry hostility), low levels of anxiety, as well as high levels of altruism and driving internality. Overall, we expect that this second cluster of moped riders would be characterized by a low risk, showing a low accident risk perception, high levels of attitudes toward safety, as well as a low frequency of risky driving behaviors and accidents involvement. Finally, we expect a third group that despite would be characterized by some personality traits related to risky driving, such as emotional instability (i.e., angry hostility and high driving anger) and low levels of altruism and internality, they would present several traits that typically predict safe driving, such as low levels of excitement-seeking and normlessness, as well as high levels of anxiety. Overall, we expect that the potential risk represented by the emotional instability and low altruism in this last cluster, may be greatly buffered by the latter traits, characterizing these moped riders as a low risk group, with high level of accident risk perception alongside with positive attitudes toward safety and low risky driving behaviors.

## Materials and Methods

### Participants and Procedures

The study relies on a sample of 1,273 Italian high school students aged from 13 to 19 years (mean age = 15.43, *SD* = 0.98), who attended the first 3 years of the high school (28% first year, 42.4% second year, 29.6% third year), distributed in different Italian regions (34.2% Northern, 23.4% Centre, 42.4% Southern) and with a valid driving license for moped. Participants were mainly males (70.4%), and they have held a valid driving license for moped for an average of 18.13 months (*SD* = 12.00). About one third of respondents (36.1%) reported daily moped riding, while the 12.8% drove 100 km or more on a weekly basis. The study was approved by the Ethics Review Board of the Department of Social and Developmental Psychology, “La Sapienza” University of Rome, and participants and their legal representatives were informed of the aims and purpose of the study, as well as their participation rights (e.g., confidentiality of responses, allowance to leave the study at any point without any consequences), in advance of data collection. Thus, written informed consent was obtained by all the participants and, for the participants under the age of 18, also by their parents.

### Procedure

The study took place in 54 high schools all over Italy. The study was firstly presented to schools (teachers and managers) and parents through informed letters sent by the PI, and then by an assistant researcher (psychologist) who collected the informed consent by the parents of minors. Therefore, a psychologist introduced by the teacher presented the study to the participants face to face during a dedicated hour of lesson, one class at a time. At this time, informed consent was collected from participants aged over 18 years old, before the data collection. In order to guarantee for the anonymity, data collection instruments did not contain information that could identify participants. Participants were asked to complete a questionnaire, to envelope it in a folder and then to place it in a collection box. Folders and boxes were provided by the researcher.

### Measures

For the purpose of this study, we used the measures listed below, which were previously translated in Italian and used in previous studies with Italian samples of drivers from different ages (i.e., Lucidi et al., 2010, 2014; Mallia et al., 2015).

#### General Personality Measures

Four facets of the Italian version (Caprara et al., 2001) of the “NEO-Personality Inventory” (Costa and McCrae, 1992) were used to evaluate general personality traits such as excitement-seeking (E5) (e.g., *I often crave excitement*), angry hostility (N2) (e.g., *I often get angry at the way people treat me*), anxiety (N1) (e.g., *I often feel tense and jittery*), and altruism (A3) (e.g., *I generally try to be thoughtful and considerate*). Each facet consisted of eight items, with responses given on five-point Likert-type scale ranging from “strongly disagree” (1) to “strongly agree” (5).

Normlessness (which refers to “the belief that socially unapproved behaviors are required to achieve certain goals,” Lucidi et al., 2014, p. 320) was assessed using the “Normlessness Scale” (Kohn and Schooler, 1983), which comprised four items (e.g., *If something works, it is less important whether it is right or wrong*), with responses made on a five-point Likert scale ranging from “strongly disagree” (1) to “strongly agree” (5).

#### Driving Related Personality Measures

The questionnaire also measured some personality characteristics specifically associated to driving. In particular, driving anger was assessed with the short version of the “Driving Anger Scale” (Deffenbacher et al., 1994), which consisted of 14 items and measured the tendency to become irritable, frustrated and angry in different traffic situations. Respondents were invited to imagine that they were experiencing a hypothetical situation (e.g., *Someone backs right out in front of you without looking, or Someone is weaving in and out of traffic*) and then they were asked to rate the extent to which they would experience anger using a five-point Likert scale, ranging from *“*I wouldn’t get angry at all” (1) to “I would get very angry” (5). Higher scores in this measure represent higher scores in anger at driving.

Furthermore, the locus of control orientation in driving was measured with the “Driving Internality” (DI, e.g., *Accidents are only the result of mistakes made by the driver*) and “Driving Externality” (DE, e.g., *Driving with no accidents is mainly a matter of luck*) Scales (Montag and Comrey, 1987). Each scale consisted of 15 items with responses given on six-point Likert-type scales ranging from “strongly disagree” (1) to “strongly agree” (6).

#### Attitudes Toward Traffic Rules

The attitudes toward traffic rules were measured with the scale developed by Iversen and Rundmo (2004). The scale measured attitudes of participants toward the infraction of traffic rules and speeding (11 items, e.g., *Many traffic rules must be ignored to ensure traffic flow*), the negligent driving of others (3 items, e.g., *I will ride with someone who speeds if that’s the only way to get home at night*) and driving after drinking (2 items, e.g., *I would never drive after drinking alcohol*). Participants were asked to rate each item on five-point Likert-type scale ranging from “strongly disagree” (1) to “strongly agree” (5), with higher scores representing a more negative attitude toward traffic safety.

#### Accident Risk Perception

Crash risk perception was assessed by two items (e.g., Lucidi et al., 2010). The first item evaluated the drivers’ subjective probability of being involved in a traffic accident relatively to their peers, the second item their level of concern about this possibility. Responses were given on rating scales from “very low” (1) to “very high” (10) for both items. The responses on each item were aggregated in a single score, with higher scores reflecting higher crash risk perception.

#### Driving Behavior and Driving Experience

Different risky behaviors were assessed through measures derived from Iversen and Rundmo (2004) study on the following dimensions:

(a) frequency of traffic rules’ violations and speeding (five items, e.g., *Break traffic rules to secure more continuous driving*); (b) frequency of reckless driving and fun riding (five items, e.g., *Drive too close to the car in front to be able to stop if it should brake*); (c) frequency of not using helmet, (two items, e.g., *Drive short distances without wearing the helmet*); (d) frequency of cautious and watchful driving (four items, e.g., *Reduce speed when you see a sign indicating danger*); (e) frequency of drinking and driving (three items, e.g., *Drive after you have been drinking more than one glass of beer or wine*). For each of the described activities, participants were requested to indicate how often they carried out or experienced it, by using a five-point Likert-type scale ranging from “never” (1) to “very often” (5).

Additionally, participants were requested to indicate how often they drive and the number of kilometers they traveled weekly over the past 3 months. Finally, they were requested to report whether they have received tickets for traffic violations (Yes vs. No) and whether they were involved in crashes with vehicle damage (Yes vs. No) and physical injury (Yes vs. No) in the past year. Table 1 reported the descriptive statistics and reliability coefficients for all the measures described above.

**Table 1:** Correlations, mean scores, SD and Cronbach’s alpha for the personality, attitudes, risk perception and driving behavior measures.

	1	2	3	4	5	6	7	8	9	10	11	12	13	14	15	16	17
1. Anxiety	–																
2. Angry hostility	0.25	–															
3. Excitement seeking	−0.21	0.15	–														
4. Altruism	0.04	−0.29	0.01	–													
5. Normlessness	−0.20	0.17	0.35	−0.21	–												
6. Driving anger	0.04	0.22	0.25	−0.02	0.24	–											
7. Driving internality	0.06	−0.09	−0.11	0.14	−0.13	−0.09	–										
8. Driving externality	−0.03	0.13	0.22	−0.01	0.31	0.18	−0.05	–									
9. Attitude toward rule violation and speeding	−0.19	0.17	0.33	−0.24	0.58	0.28	−0.25	0.31	–								
10. Attitude toward careless driving of others	−14	0.09	0.26	−0.22	0.37	0.13	−0.18	0.14	0.48	–							
11. Attitude toward drinking and driving	−0.09	0.12	0.19	−0.22	0.28	0.10	−0.16	−0.01	0.33	0.41	–						
12. Risk perception	0.24	0.09	−0.05	0.03	−0.06	0.06	0.08	0.00	−0.10	−0.07	−0.02	–					
13. Violations of traffic rules/speeding	−0.21	0.14	0.39	−0.17	0.42	0.27	−0.14	0.19	0.53	0.30	0.24	−0.07	–				
14. Reckless driving/fun riding	−0.10	0.19	0.22	−0.28	0.36	0.20	−0.11	0.11	0.43	0.35	0.33	−0.01	0.57	–			
15. Not using helmet	−0.11	0.13	0.26	−0.14	0.32	0.13	−0.02	0.14	0.33	0.31	0.29	−0.01	0.37	0.49	–		
16. Cautious and watchful driving	0.14	−0.04	−0.11	0.23	−0.26	−0.07	0.14	−0.01	−0.29	−0.23	−0.22	0.13	−0.20	−0.25	−0.12	–	
17. Drinking and driving	−0.17	0.16	0.27	−0.23	0.37	0.18	−0.16	0.05	0.42	0.39	0.48	−0.06	0.45	0.57	0.43	−0.23	–
Mean	2.98	2.91	3.58	3.71	2.62	3.62	2.55	2.72	2.76	1.89	1.67	5.27	3.08	2.23	2.03	3.43	1.99
(SD)	(0.63)	(0.59)	(0.69)	(0.58)	(0.82)	(0.59)	(0.69)	(0.61)	(0.73)	(0.89)	(0.95)	(2.06)	(1.05)	(0.90)	(1.23)	(0.85)	(1.10)
Skewness	0.05	0.19	−0.37	−0.35	0.31	−0.19	−0.17	0.02	0.31	0.97	1.75	−0.02	0.01	0.93	1.03	−0.51	0.98
Kurtosis	0.09	0.00	−0.14	0.24	−0.21	0.05	0.14	−0.01	−0.20	0.48	2.54	−0.49	−0.82	0.57	−0.10	0.02	0.02
Alphas	0.60	0.52	0.63	0.62	0.60	0.74	0.78	0.69	0.78	0.63	0.84	0.42	0.87	0.81	0.76	0.66	0.80

*All the correlation coefficients are statistically significant at least at a p-level of 0.05, apart from underlined coefficients.*

### Data Analysis

Firs of all, in order to group together individuals whose characteristics are similar, a cluster analysis was performed through the “Classify” Package of SPSS 22.0 and using the squared Euclidean distance measure. The variables used to identify subtypes of young moped riders were the scores obtained at the personality measures (general and specific) used in previous studies (i.e., Lucidi et al., 2010): anxiety, angry hostility, excitement-seeking, altruism, normlessness, driving anger, driving internality, and driving externality. Participant who reported missing data on at least one of these variables, were excluded from the cluster analysis.

Standardized scores (*Z*-scores) of the key personality variables were computed and used for cluster analysis in order to overcome the issue of comparing Euclidean distances based on different measurement scales (Everitt, 1993). In particular, we initially employed a hierarchical cluster analysis, using a Ward’s method of linkage and a squared Euclidean distance, to identify the number of cluster groups according to the parameter of the increment of the merger coefficients (Fabbris, 1997). At the point of marked flattening of the graph, the subsequent mergers of cluster portrayed no new information. Although the hierarchical clustering method is advantageous for determining the number of clusters, it does not allow the determination of the most optimal cluster solution pertaining to between-cluster heterogeneity. This is because the method cannot separate clusters created in previous steps. Thus, following the recommendations (Milligan and Sokol, 1980) concerning a K-means non-hierarchical method using centroids from the hierarchical cluster analysis (i.e., the cluster center means), we employed a K-means method to identify the most optimal three clusters solution that emerged from the data. Finally, a multivariate analysis of variance (MANOVA) was carried out using the raw scores of the key personality variables used in the cluster analysis (i.e., anxiety, angry hostility, excitement-seeking, altruism, normlessness, driving anger, driving internality and driving externality) as dependent variables and the cluster membership (Cluster A vs. Cluster B vs. Cluster C) as the independent variable, with the aim to confirm the differences on key personality variables between the groups generated by the cluster analysis.

The external validation of the cluster solution, was rather obtained by using significance tests on relevant criteria variables that were not used to generate the cluster solution (Alivernini et al., 2016). In particular a second multivariate analysis of variance (MANOVA) was utilized to examine whether the clusters identified differed on the raw scores of the three subscales measuring drivers’ attitudes, of the accident risk perception scale, and of the five subscales measuring riders’ driving behaviors. LSD *post hoc* tests were also used to determine which clusters differed from each other in their mean scores on these variables. In order to measure the strength of the association between the clusters and the various key dependent variables, the η_p_^2^ was calculated. Cramer V test was used to examine whether the clusters identified differed in dichotomous variables related to driving habits and experience, such as driving every-day (Yes vs. No), driving more than 100 km per week (Yes vs. No), having received at least one ticket (Yes vs. No), and being involved in at least one accident with vehicle damage (Yes vs. No) and/or physical injury (Yes vs. No) in the last year. Overall, missing data were treated listwise for all the multivariate analyses, while for the bivariate correlations a pairwise approach has been used.

## Results

### The Cluster Solution and Cluster Profiles

**FIGURE 1:**
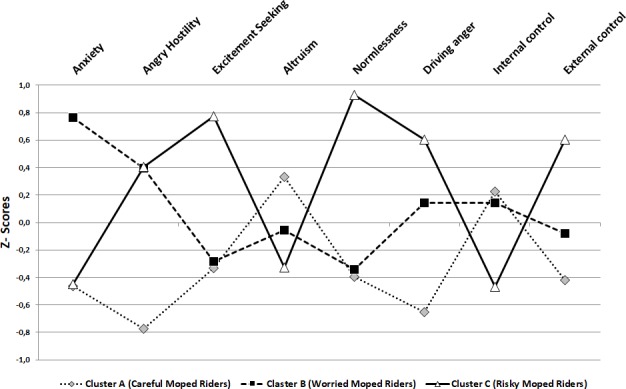
Profile plot of the three clusters of moped riders identified.

Seven participants reported missing data on at least one of the personality variables, so the cluster analysis was carries out on 1,266 participants. An examination of the merger coefficients’ graph and of the dendrogram (see Supplementary Appendix S1) indicates a three-cluster solution. In the subsequent non-hierarchical clustering procedure, we identified the most optimal three-cluster solution that emerged from the data. The final centers for each cluster and the distances between the final cluster centers are reported in Supplementary Appendix S2. The standardized (*Z*-scores) cluster means of the variables generated by the K-means analysis on the three-cluster solution are showed in Figure 1.

The moped riders grouped in Cluster A showed low rates in driving anger, anxiety, angry-hostility and excitement seeking. This pattern of personality scores indicates that the moped riders of this group are quiet and stable from an emotional point of view. Moreover, high levels of altruism and low levels of normlessness suggest that they seriously take into consideration the rules and traffic regulations and give attention to others on the road. Finally, these moped riders reported higher levels of internal driving control than on external driving control, this representing their beliefs that crashes are primarily the result of drivers’ mistakes, and therefore there are preventable through own riding behavior. Based on the description above, these types of riders have been called “careful moped riders.”

Moped riders in Cluster B are characterized by high levels of anxiety, angry hostility and driving anger, and low levels of excitement seeking, normlessness and altruism. This pattern suggests that although this group of riders may respect traffic rules (i.e., low normlessness) and avoid intentional risky behavior (i.e., low excitement seeking), their high level of emotional instability (i.e., high angry hostility and driving anger) and their lack of concern for others (i.e., low altruism) may make them potentially at risk on the road. However, similar to the moped riders of Cluster A, this group showed relatively higher levels of internal driving control than on external driving control, reflecting internal/driver-centered attributions for what happens on the road. At the same time, the high level of anxiety may stimulated worries about the possible consequences of their actions while driving, increasing the risk perception and the attention to not commit risky behaviors. Thus, the moped riders in Cluster B were labeled “worried moped riders.”

High rates of normlessness and low levels of altruism characterized riders in Cluster C, suggesting that moped riders in this cluster are less likely to respect the rules and to be concerned about others. Furthermore, they reported high levels of excitement seeking and low levels of anxiety, suggesting that they enjoy doing extreme actions without being scared or worried about possible consequences. Cluster C moped riders also showed low levels of tolerance to frustration in various traffic situations, as suggested by high rates of driving anger and angry hostility. Finally, they also reported higher rates of external driving control than of internal driving control, meaning that for them, accidents are primarily due to external causes, for instance related to bad roads or mechanical problems or simply to bad luck and therefore, they are not preventable through their own self-regulated behavior. Based on this description, and the fact that the riders in this group are expected to be at high risk for traffic violations and accidents, they were defined, “risky moped riders.”

Overall, as expected, the three clusters described above resulted significantly different on the key personality variables included in the cluster analysis (Wilkin’s Lambda _(16,2512)_ = 0.214; *p* < 0.001; $\eta_{p}^{2}$ = 0.55). The mean raw scores of these personality measures for moped riders in each of the three clusters identified and the univariate tests are shown in Table 2.

**Table 2:** Cluster differences on the raw scores of personality (general and specific) measures included in cluster analysis.

	Cluster groups				
	Cluster A	Cluster B	Cluster C	F	$\eta_{p}^{2}$
Anxiety	2.68 (0.49)^B^	3.47 (0.48)^AC^	2.69 (0.57)^B^	340.84^∗∗^	0.35
Angry hostility	2.46 (0.44)^BC^	3.15 (0.47)^A^	3.16 (0.58)^A^	286.98^∗∗^	0.31
Excitement seeking	3.36 (0.66)^C^	3.39 (0.63)^C^	4.11 (0.48)^AB^	196.43^∗∗^	0.24
Altruism	3.90 (0.53)^BC^	3.68 (0.52)^AC^	3.52 (0.65)^AB^	47.35^∗∗^	0.07
Normlessness	2.29 (0.70) ^C^	2.34 (0.61) ^C^	3.38 (0.69)^AB^	325.50^∗∗^	0.34
Driving anger	3.23 (0.51)^BC^	3.70 (0.51)^AC^	3.97 (0.51)^AB^	218.47^∗∗^	0.26
Driving internality	2.70 (0.60)^C^	2.65 (0.66)^C^	2.22 (0.74)^AB^	59.56^∗∗^	0.09
Driving externality	2.47 (0.57)^BC^	2.67 (0.54)^AC^	3.09 (0.56)^AB^	125.91^∗∗^	0.17

*^∗∗^p < 0.001. ^A,B,C^Cluster groups that result significantly different at LSD post hoc test (p < 0.001).*

### Attitudes, Risk Perception, and Driving Behaviors of Moped Riders in Each of the Cluster Profiles

Results of the comparisons made between the three groups of moped riders on descriptive characteristics are shown in Table 3, results on the comparisons made on driving-related outcome measures are shown in Table 4.

**Table 3:** Cluster differences on descriptive measures.

	CLUSTER				
	Cluster A Careful moped riders	Cluster B Worried moped riders	Cluster C Risky moped riders	Cramer V or F	*p*-level
% of the total	34.4%	37.3%	28.3%		
% Males	74.0%	61.4%^C^	77.7%^B^	0.15	<0.001
Mean age	15.42 (1.02)^C^	15.32 (0.91)^C^	15.58 (1.00)^AB^	6.81	0.001
Months that they have license to drive moped	18.07 (11.25)	17.22 (11.49)	19.44 (13.51)	2.82	0.06
Driving every-day	33.7%	32.6%^C^	42.5%^B^	0.90	0.007
Driving more than 100 km a week	11.2%^C^	9.3%^C^	18.2%^B^	0.11	<0.001

*^A,B,C^ Cluster groups that result significantly different at LSD post hoc test (p < 0.001).*

**Table 4:** Cluster differences on the raw scores of driving outcome measures.

	Cluster groups					
	Cluster A Careful moped riders	Cluster B Worried moped riders	Cluster C Risky moped riders	Cramer V or F	*p*-level	$\eta_{p}^{2}$
Received at least one ticket	13.3%	11.4%^C^	19.8%^B^	0.10	0.002	–
Had at least one accident with only vehicle damage	11.7%^C^	11.9%^C^	17.3% ^AB^	0.07	0.03	–
Had at least one accident as driver with physical injury	7.1%^C^	7.2%^C^	14.5%^AB^	0.12	<0.001	–
**Drivers’ attitude toward**						
Rule violation and speeding^2^	2.48 (0.64) ^BC^	2.57 (0.62) ^AC^	3.34 (0.66) ^AB^	205.43	<0.001	0.25
Careless driving of others^2^	1.69 (0.74)^C^	1.72 (0.79) ^C^	2.39 (0.98) ^AB^	82.25	<0.001	0.12
Drinking and driving^2^	1.48 (0.82) ^C^	1.58 (0.87) ^C^	2.04 (1.09)^AB^	37.10	<0.001	0.06
Risk perception^1^	4.99 (1.95)^B^	5.63 (2.11)^AC^	5.14 (1.80)^B^	11.68	<0.001	0.02
**Driving behaviors**						
Violations of traffic rules/speeding^3^	2.79 (0.97)^C^	2.80 (0.93)^C^	3.81 (0.94)^AB^	142.80	<0.001	0.19
Reckless driving/fun riding^3^	1.95 (0.71)^BC^	2.14 (0.81)^AC^	2.72 (1.04)^AB^	81.65	<0.001	0.12
Not using helmet^3^	1.78 (1.04)^C^	1.83 (1.09)^C^	2.62 (1.41)^AB^	59.84	<0.001	0.09
Cautious and watchful driving^4^	3.51 (0.79)^C^	3.55 (0.79)^C^	3.17 (0.94) ^AB^	24.29	<0.001	0.04
Drinking and driving^3^	1.70 (0.89)^C^	1.80 (0.96) ^C^	2.61 (1.25) ^AB^	91.45	<0.001	0.13

*^A,B,C^ Cluster groups that result significantly different at LSD test (p < 0.001). ^1^ Range 1–10: a high score on the scale indicate high perception of risk to have a traffic accident. ^2^ Range 1–5: a high score on the scale reflects a negative attitude toward traffic safety. ^3^ Range 1–5: a high score indicates risky driving behavior. ^4^ Range 1–5: a high score indicates a safe driving behavior.*

The three clusters differed on age, being the moped riders of the risky cluster slightly older than riders of the other two clusters. The three clusters did not differ in the number of months they have held a driver’s license; on the other hand the “risky moped riders” drove daily more frequently and were more likely to drive more than 100 km a week than the “worried moped riders” were. Furthermore, the higher risk for the risky moped riders, compared with the other two groups of riders (i.e., “careful moped riders” and “worried moped riders“), was confirmed by overall differences in several driving-related outcomes such as their past driving experience, their risk perception, their attitudes toward traffic safety and their self-reported risky driving behaviors (Wilkin’s Lambda _(18,2446)_ = 0.664, *p* < 0.001; $\eta_{p}^{2}$ = 0.18).

A significantly larger percentage of moped riders in Cluster C (i.e., “risky moped riders”), had received at least one ticket with respect to moped riders defined as “worried,” and were involved in at least one accident with vehicle damage and with physical injury if compared with the other two clusters. Furthermore, the risky moped riders showed the most negative attitudes toward traffic safety, and, despite they were highly involved in accidents, they showed lower accident risk perception than worried moped riders. With respect the self-reported risky driving behavior, the moped riders in Cluster C (i.e., “risky moped riders”) reported significantly more frequent involvement in violations of traffic rules and speeding, more reckless driving and fun riding, driving without the helmet, and more drunk driving as compared to the moped riders in the other two clusters. Accordingly, the moped riders in Cluster C reported a lower frequency of safe driving behaviors such as cautious and watchful driving than worried and careful riders.

Moped riders in Cluster A, (i.e., “careful moped riders”) demonstrated an opposite profile with respect to moped riders of Cluster C. In particular, a significant tinier percentage of them were involved in accidents with vehicle damage and with physical injury, they reported more positive attitudes toward traffic safety than moped riders of Cluster C, and a lower level of risk perception than moped riders of Cluster B. Furthermore, moped riders in Cluster A reported a lower frequency of risky driving behaviors (e.g., traffic rule violations, drink and driving, etc.) and a higher frequency of safe behaviors (e.g., cautious and watchful driving) at the wheel than the moped riders in Cluster C (i.e., risky moped riders).

Moped riders of Cluster B (i.e., “worried moped riders”) showed a low risk profile, very similar to the careful drivers’ profile in terms of driving experience, attitudes toward safety and driving behaviors. In fact, within the worried riders, a smaller percentage reported to be involved in accidents as compared to risky moped riders. Furthermore worried riders showed also higher positive attitudes toward traffic safety and a lower frequency of risky driving behaviors as compared to moped riders in Cluster C (i.e., “risky moped riders”). However, it is noteworthy that the moped riders in Cluster B reported the highest level of risk perception to be involved in an accident. Finally, the gender distribution was more balanced (i.e., 61.4% of males) within the worried moped riders than within the other two subgroups (i.e., 74% and 77.7% of males, respectively, for careful and risky moped riders).

## Discussion

The present study responds to the need to better understand adolescent moped riders behavior since they represent a high-risk group for road traffic injuries and fatalities in Europe (e.g., Brandau et al., 2011; Vlahogianni et al., 2012; Theofilatos and Yannis, 2015). Following previous studies on adolescent moped riders in different European countries (e.g., Brandau et al., 2011; Marengo et al., 2012), the present study investigated, within a large sample of Italian adolescent moped riders, sub-types of riders based on diverse personality traits, and compared them across a range of psychological and behavioral measures including attitudes toward safe driving, self-reported risky driving behaviors (e.g., rule’s violations and speeding, not using helmet, drinking and driving, etc.), and self-reported issued traffic tickets and crash involvement.

Our findings showed that the adolescent riders of our sample can be grouped in three distinct clusters, which are related to different personality traits as well as to different attitudes and behaviors (as in the case of risky moped drivers). The analysis of the different personality characteristics led to the grouping of moped riders as careful, worried and risky. Importantly, in accordance with previous research (i.e., Ulleberg and Rundmo, 2003; Lucidi et al., 2010; Marengo et al., 2012), the present findings lend support to the notion that while some personality characteristics are associated to risky driving tendencies among moped riders, other personality characteristics may act as protective factors. The clusters identified in the present study resembled substantially those identified by Brandau et al. (2011) and Marengo et al. (2012); thus, showing that some of the measured psychological characteristics are associated with risky driving beliefs and behaviors across studies and independently of sample sizes nationality, and research methods used.

In particular, negative attitudes toward traffic safety were higher in those moped riders who reported higher levels of emotional instability (i.e., high rates of driving anger and angry-hostility) and excitement seeking, lower levels of altruism, and higher driving externality (i.e., the belief that accidents depends for the most part on bad luck or on external causes uncontrolled by the driver). The combination of such attitudes, risk perceptions and personality characteristics in these mopeds riders was associated with several indicators of risky driving behaviors, such as higher self-reported frequency of traffic rules’ violations and speeding, reckless driving and fun riding, not wearing helmet while driving, as well as drinking and driving. Not surprisingly, adolescents in the “risky moped drivers” cluster were more likely to receive at least one traffic ticket and to be involved in a car crash with vehicle damage and/or physical injury in the last year, as compared to adolescent moped riders in the other two clusters. The profile of the moped riders identified here as at higher risk resembles the pattern of personality traits identified as at risk by Marengo et al. (2012) in adolescents moped riders and by Lucidi et al. (2010) in novice car drivers.

Although some moped riders in our study displayed similar personality characteristics with risky moped riders (i.e., high emotional instability, low altruism), they reported more positive attitudes toward traffic safety, and lower frequency of risky driving behaviors. These “worried moped riders” were characterized by the highest levels on anxiety, and by the highest levels of risk perception to be involved in an accident. In other words, high levels of anxiety in this group may buffer the relationship between emotional instability and risky attitudes and driving. In terms of a process, being anxious and worried to be involved in a crash could attenuate the effects of emotional instability on risky driving decision-making (e.g., deciding to violate traffic rules or ride the moped without a helmet). The present study had mainly a descriptive purpose and did not directly tested this process, therefore future studies addressing this issue are strongly recommended. Overall, the profile of “worried moped drivers” is very similar to the profiles identified by Lucidi et al. (2010) and Marengo et al. (2012). The only difference between the profile identified by Marengo et al. (2012) and the worried drivers of this study, is that in our study worried riders are characterized by higher internal driving control, whereas Marengo et al. (2012) riders showed higher external driving control. This difference was probably due to the fact that more than half of the adolescents in the Marengo et al. (2012) study did not have a direct experience in moped riding, so this may have fostered the perception of external driving control.

In any case, our findings related to worried moped riders have an applied value for safe driving interventions because they seem to suggest that fear appeals or message framing (emphasizing the “losses” of risky driving) may be effective in risk communication by eliciting greater fear of accidents and emphasizing the personal relevance and susceptibility for crash involvement and/or traffic injury. However, more research on this issue is needed, especially in the light of the findings by Carey et al. (2013) who meta-analyzed the impact of fear appeals on driver behavior: although fear appeals increased fear arousal, they did not have the desired impact on actual driving behavior. According to some authors (e.g., Stephens and Groeger, 2009) anxious drivers could be more likely to drive cautiously and comply with traffic rules, probably also because of a lack of confidence about their driving ability. From this perspective, it is plausible that, especially in very young drivers, the seemingly protective effect of anxiety can fade over time as young drivers become more experienced, and this change may be followed by changes in the respective attitudinal and behavioral profile of worried moped riders.

Furthermore, according to our hypothesis, moped riders with higher levels of emotional stability (i.e., low driving anger, low anger hostility), low anxiety, low scores on excitement seeking and high scores on altruism and driving internality appeared as a low risk group (labeled “careful drivers”). In particular these traits were associated with more positive attitudes toward traffic rules and with a lower frequency in all the indicators of risky driving behavior and also in self-reported crash involvement. This was in line with the “personality-attitudes” model introduced by Ulleberg and Rundmo (2003). The “careful” profile identified in this study was also similar to the pattern of personality measures identified by previous study on moped riders (Marengo et al., 2012) and on novice car drivers (Lucidi et al., 2010).

The set of measures of personality, safe driving attitudes and risky driving used in the present study had never been used in previous research on moped riders. On the contrary, these measures were virtually identical to those used in previous research on car drivers (Lucidi et al., 2010), aged between 18 and 23 years, allowing us to draw an indirect comparisons between novice car drivers and adolescent moped riders. To ride a moped and to drive a car at a younger age are actions that need to be considered very differently, since drivers are exposed to different accident risks (e.g., Zambon and Hasselberg, 2006), require different skills and abilities such as hazard perception (e.g., Horswill and Helman, 2003; Rosenbloom et al., 2011), and bring drivers to experience different levels of aggression in traffic (e.g., Rowden et al., 2016) or of risky behaviors such as errors, lapses and violations (Topolšek and Dragan, 2015). Specifically, riding a moped is a complicated task that requires specific attentional and individual skills, and riders’ perceptions and attitudes are important as they reflect their actual behavior on the road (Theofilatos and Yannis, 2015). Taken altogether, the results of present study clearly showed that the identified sub-types of moped riders substantially resembled the sub-types of moped riders identified by Marengo et al. (2012) and of novice car drivers identified by Lucidi et al. (2010). In other words, despite the specificities of the vehicle used (i.e., car vs. moped) the ways that personality characteristics are grouped and, consequently, are associated with risky driving attitudes and behavior appeared highly similar.

### Practical Applications

Our study provided empirical support to the fact that the personality characteristics are consistently associated with attitudes toward traffic safety and risky driving behaviors in moped riders as in car drivers. This may suggest that an intervention designed to tackle risky driving messages on the basis of personality sub-types in young drivers can impact risky behaviors on diverse young populations, from adolescent moped riders to novice young adult drivers. By no means, this is not an assertion of an “one size fits all” approach, but rather a call for more concerted evidence-based interventions to reduce the risk for road fatalities by tackling specific psychological and behavioral factors. The characteristics of the highest risk group identified in the present study as well as in previous research involving both moped riders (e.g., Marengo et al., 2012) and car drivers (i.e., Ulleberg and Rundmo, 2003; Lucidi et al., 2010) suggested that such educational interventions could focus on the emotional characteristics (e.g., anger hostility levels) of the drivers. As Lucidi et al. (2010, p. 1695) claim “angry reactions in driving situations, for example, may trigger responses such as traffic rule violations and speeding, especially in young novice drivers who demonstrate high levels of excitement-seeking and normlessness.” Furthermore, our results are in line with the evidences that educational interventions may benefit from a focus on emotional regulation on the road (Deffenbacher, 2016). Different studies in the behavioral sciences and neuroscience have shown that poor emotion and self-regulation were associated with a wide range of risk-taking and health compromising behaviors especially among young people (Magar et al., 2008; Steinberg, 2008; Spano et al., 2018). A large number of studies have shown that interventions that include physical and cognitive relaxation were effective in reducing driving anger and aggression in angry drivers (for a review, see Deffenbacher, 2016). For example, Deffenbacher et al. (2000) showed that a short-term intervention with the inclusion of relaxation coping skills or both cognitive and relaxation skills decreased traffic-related anger among drivers with higher levels of anger. Finally, based on our findings and those of previous studies (i.e., Lucidi et al., 2010) the association between emotional factors (e.g., anxiety), traffic safety attitudes and risky driving behavior seemed to emerge as early as adolescence. Therefore, interventions that will tackle the emotional and self-regulation aspects of driving could have an impact early on, as soon as or even before young people engaged in actual driving (e.g., moped riding) – thus, allowing for primary interventions on safe driving.

### Limitations

The results of this study need to be interpreted in light of some limitations. Firstly, we used a cross-sectional design which may have limited the validity of the clusters identified. However, given that personality characteristics are stable over time, we can still claim that the attitudes and self-reported driving behaviors were improbably to have anticipated and affected personality traits. Nevertheless, prospective studies should be conducted in order to support the predictive validity of the driver sub-types identified in the present study, as well as to overcome the issue of reverse causality. Further, future studies aiming to replicate our study in different samples are also needed in order to provide additional evidences for the generalizability of our conclusions (Alivernini et al., 2016; Alivernini and Manganelli, 2015). Furthermore, within the limitations, it is worth to mention that the present study mainly aimed to describe how personality traits tended to group within a sample of moped drivers, using attitudes and behavioral outcomes merely as explicative variables, in order to validate these groups in view of past evidences. Future studies directly aimed to study the link between personality traits, attitudes and behaviors in moped drivers, thus, are strongly recommended. Another limitation of the present study is the use of self-reported driving behavior, which may have been affected by social desirability or recall biases, undermining the reliability of the study. However, the fact that the questionnaires were answered anonymously, decreased this risk (Lajunen and Summala, 2003). However, future studies that use more objective measures of driving behavior, such as for example driving simulator and/or external evaluation of road driving, are needed. Furthermore, it should be noted that the power of moped riders’ sub-types to predict various driving-related outcome measures is limited. A final limitation of the study it is represented by the differences in terms of sample size between male (70.4%) and female (29.6%). However, this disproportion correctly represent the distribution of moped riders in Italy with a larger number of male than women riding during those ages (i.e., 4.8 per 100 male inhabitants versus 2.4 per 100 female inhabitants)^1^.

Despite those limitations, these results confirmed the conclusions of the Organisation for Economic Co-operation and Development [OECD], 2006 report, that the associations between personality characteristics and accident involvement in young drivers may be limited, but still consistent across studies. After all, research on the psychological and behavioral aspects of risky driving is not panacea for all crash-related risk factors, but rather a useful approach to better understand one of the most important component of crash involvement, that is, the driver’s behavioral outlook.

## Conclusion

The study identified three subgroups of moped riders (risky, worried, and careful) characterized by different patterns of personality traits, and of self-reported risky driving behaviors, attitudes toward traffic safety, risk perception, and self-reported accident involvement. The personality and behavioral characteristics of these three sub-types of moped riders substantially resembled those identified by studies with vehicle drivers, showing that specific combinations of personality characteristics are associated with risk driving tendencies and behaviors both in young moped riders and novice car drivers. The results of the present study supported that safe driving interventions should tackle risky driving beliefs and behavioral tendencies in young moped riders and car drivers by tailoring their messages according to the personality sub-types of the target groups.

## Author Contributions

All the authors substantially have equally contributed to the development and preparation of the manuscript. Furthermore, all authors have approved the final version of the manuscript. Finally, the authors have agreed to be accountable for all aspects of the manuscript in ensuring that questions related to the accuracy or integrity of any part of it are appropriately investigated and resolved.

## Conflict of Interest Statement

The authors declare that the research was conducted in the absence of any commercial or financial relationships that could be construed as a potential conflict of interest.

## Footnotes

1 http://dati.istat.it

## Supplementary Material

APPENDIX S1 Dendrogram of the hierarchical cluster analysis.

APPENDIX S2 Centers for the three clusters and distances between the final cluster centers.
