# Supplementary figures and images for: Riding the Adolescence: Personality Subtypes in Young Moped Riders and Their Association With Risky Driving Attitudes and Behaviors
Source: Front Psychol. 2019 Feb 18;10:300. doi: 10.3389/fpsyg.2019.00300 (PMC6387963; doi:10.3389/fpsyg.2019.00300)

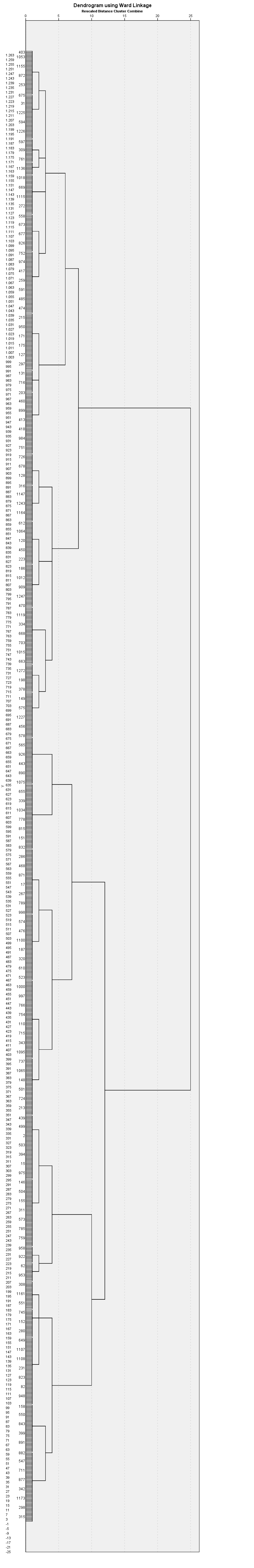

Supplement: APPENDIX S1 — Dendrogram of the hierarchical cluster analysis. [file Image_1.TIF]
