# Supplementary material for: Riding the Adolescence: Personality Subtypes in Young Moped Riders and Their Association With Risky Driving Attitudes and Behaviors
Source: Front Psychol. 2019 Feb 18;10:300. doi: 10.3389/fpsyg.2019.00300 (PMC6387963; doi:10.3389/fpsyg.2019.00300)
Supplement: APPENDIX S2 — Centers for the three clusters and distances between the final cluster centers. [file Table_1.DOCX]

Appendix 2

| **Final Cluster Centers** | | | |
| --- | --- | --- | --- |
|  | Cluster | | |
|  | 1 | 2 | 3 |
| Anxiety | -,47028 | ,77733 | -,43876 |
| Angry hostility | -,74953 | ,39917 | ,43382 |
| Excitement seeking | -,24517 | -,33224 | ,76433 |
| Altruism | ,35748 | -,08307 | -,35197 |
| Normlessness | -,40193 | -,32998 | ,96926 |
| Driving anger | -,62193 | ,13352 | ,63077 |
| Driving Internality | -,37084 | -,09794 | ,61029 |
| Driving Externality | ,21069 | ,13912 | -,46969 |

| **Distances between Final Cluster Centers** | | | |
| --- | --- | --- | --- |
| Cluster | 1 | 2 | 3 |
| 1 |  | 1,932 | 2,793 |
| 2 | 1,932 |  | 2,358 |
| 3 | 2,793 | 2,358 |  |
